# Recurrent falls as the presentations of Gitelman syndrome in an octogenarian

**DOI:** 10.18632/aging.206216

**Published:** 2025-03-04

**Authors:** Chien-Yao Sun, Shang-Han Wu, Chia-Ter Chao, Shih-Hua Lin

**Affiliations:** 1 Department of Geriatrics and Gerontology, National Cheng Kung University Hospital, College of Medicine, National Cheng Kung University, Tainan 704, Taiwan; 2 Institute of Allied Health Sciences, College of Medicine, National Cheng Kung University, Tainan 701, Taiwan; 3 Department of Medicine, University of California San Francisco, San Francisco, CA 94143, USA; 4 Department of Geriatrics and Gerontology, College of Medicine, National Cheng Kung University, Tainan 701, Taiwan; 5 Department of Internal Medicine, National Cheng Kung University Hospital, College of Medicine, National Cheng Kung University, Tainan 704, Taiwan; 6 Division of Nephrology, Department of Internal Medicine, National Taiwan University College of Medicine, Taipei 100, Taiwan; 7 Division of Nephrology, Department of Internal Medicine, National Taiwan University Hospital, Taipei 100, Taiwan; 8 Graduate Institute of Toxicology, National Taiwan University College of Medicine, Taipei 100, Taiwan; 9 Graduate Institute of Medical Education and Bioethics, National Taiwan University College of Medicine, Taipei 100, Taiwan; 10 Division of Nephrology, Department of Internal Medicine, Min Sheng General Hospital, Taoyuan City 330, Taiwan; 11 Division of Nephrology, Department of Internal Medicine, National Defense Medical Center, Taipei 104, Taiwan

**Keywords:** fall, Gitelman syndrome, hypokalemia, older adults

## Abstract

Gitelman syndrome (GS) is the most common hereditary renal tubular disorder, with a higher carrier frequency among Asians often overlooked in older adults. Electrolyte imbalances, such as those seen in GS, are crucial considerations for older adults experiencing recurrent falls. We described an 83-year-old diabetic female on metformin, who was admitted due to recurrent falls with the preceding dizziness and palpitations when standing. She had the history of chronic hypokalemia and hypomagnesemia on regular potassium (K^+^) and magnesium (Mg^++^) supplementation for 10 years and gout-like arthritis episodes over her shoulder and ankle joints. Her consciousness was alert with normal blood pressure but reduced tendon reflex over bilateral knees. Pertinent laboratory findings included hypokalemic (K^+^ 2.2 mmol/L) with metabolic alkalosis and high urine K^+^ excretion, hypomagnesemia (1.1 mg/dl) with hypermagnesuria, but hypocalciuria (UCa/Cr ratio 0.01 mg/mg), high urine salt excretion, and hyperreninemia. X-ray of bilateral knees and shoulders demonstrated typical chondrocalcinosis with dense calcification band in the joint space. Targeted Sanger sequencing confirmed GS, identifying a biallelic homozygous deletion mutation (2881-2 delAG) in the exon 24 of *SLC12A3* gene as the potential causes of recurrent falls. After aggressive electrolytes correction, her potassium and magnesium levels stabilized, and the patient did not experience further falls. This case, probably the oldest documented patient with GS emphasizes the importance of recognizing atypical presentations of GS in older adults. Careful evaluation and management of electrolyte disturbances in this population may prevent fall recurrence and complications.

## INTRODUCTION

In older adults, hypokalemia is prevalent, impacting membrane potential and disrupting excitable tissues (e.g., nerve, muscle), leading to paresthesia, cardiac arrhythmia, and potentially fatal outcomes [1]. Common causes of hypokalemia in older adults include diuretic misuse, gastrointestinal potassium loss, and less commonly, inherited or acquired autoimmune tubulopathies.

Gitelman syndrome (GS), the most common hereditary renal tubular disorder, has a prevalence of about 1 in 40,000 worldwide [2]. This disorder stems from mutations in the *SLC12A3* gene, located on chromosome 16q13, which encodes the thiazide-sensitive Na-Cl cotransporter (NCC). To date, over 400 distinct mutations have been identified [3], encompassing missense [3–6], splice-site [3, 4], nonsense [3, 4], and frameshift mutations [7], as well as large genetic rearrangements such as duplications [7] and deletions [5, 7]. Genetic diagnostic approaches, including Sanger sequencing [3–7], multiplex ligation-dependent probe amplification (MLPA) [4, 7], and whole genome sequencing [5], have significantly expanded our understanding of the mutation spectrum of the *SLC12A3* gene. This includes pathogenic NCC mutations affecting exons [3–7], exon-intron boundaries [4], intronic [7] and flanking regions [3]. GS exhibits a heterozygous carrier frequency of approximately 1 in every 15.6 cases, with the prevalence among Asians reaching up to 10.3 per 10,000 individuals [8]. A study by Hsu et al. involving 500 unrelated children from the Taipei Children Heart Study identified a relatively high prevalence of heterozygous NCC mutations at 2.9%, consistent with findings from Japan and other Chinese studies [9]. However, lower frequencies have been observed in Western populations, such as 1% in Sweden and Italy and 0.5% in the Framingham Heart Study [9, 10]. Clinical manifestations of GS, including intermittent muscular weakness, tetany, fatigue, and joint pain [11], primarily result from the impaired NCC function, leading to a reduced sodium chloride (NaCl) reabsorption and subsequent hypokalemia, hypocalciuria, and hypomagnesemia. Early genetic testing, facilitated by the identification of biallelic mutations in *SLC12A3*, aids in accurate diagnosis, patient education, and family screening. Despite its prevalence, GS is often overlooked in older adults due to demographic biases, and there is necessity for increased awareness across all age groups.

Limited literature exists on genotyping-based diagnoses of GS in older patients [11]. Contributing factors include genotyping costs, varied biochemical and clinical manifestations in older adults, and the limited GS awareness among geriatricians. We highlight a case of an older woman presenting with recurrent falls and electrolyte imbalances. Subsequent workup, including an in-depth genetic analysis pinpointed a homozygous mutation in the *SLC12A3* gene, confirming the diagnosis of GS. Advances in sequencing technology greatly enhance precise management of hereditary tubulopathies in older patients.

## Case presentation

An 83-year-old woman presented to our geriatric clinic after recurrent falls. Her daughter noted balance loss during prayer or after using the restroom without loss of consciousness or convulsions. The patient experienced dizziness and palpitations preceding the falls, along with persistent headache, thirst, and a sensation of head ‘fullness’. In the past two months, there was a notable decline in her activity level, requiring assistance for basic tasks and walker use. Despite a good appetite and preference for salty foods, she lost significant weight, dropping from 45 to 32 kilograms over a year. She was admitted for a detailed evaluation.

According to her daughter, the patient was a non-literate homemaker with no significant developmental milestone deficits in sports or work during her younger years. She had a strong craving for salty snacks, despite her consistently short and slender stature. The patient’s medical history included diabetes mellitus, managed with metformin (1000 mg twice daily) and a glycated hemoglobin of 6.5%. She experienced occasional gout-like arthritis over her shoulder and ankle joints. A decade earlier, she was diagnosed with chronic hypokalemia and hypomagnesemia, for which she received oral supplements. Despite treatment, she remained to have muscle pain, stiffness, and general fatigue, leading to frequent hospital visits. She underwent a subtotal gastrectomy for peptic ulcer in her twenties. Her family history was notable for hypothyroidism in her oldest son, minor thalassemia in a daughter, and hypertension in her youngest son, without significant findings of electrolyte abnormalities or kidney diseases among other family members (Supplementary Figure 1).

**Figure 1:**
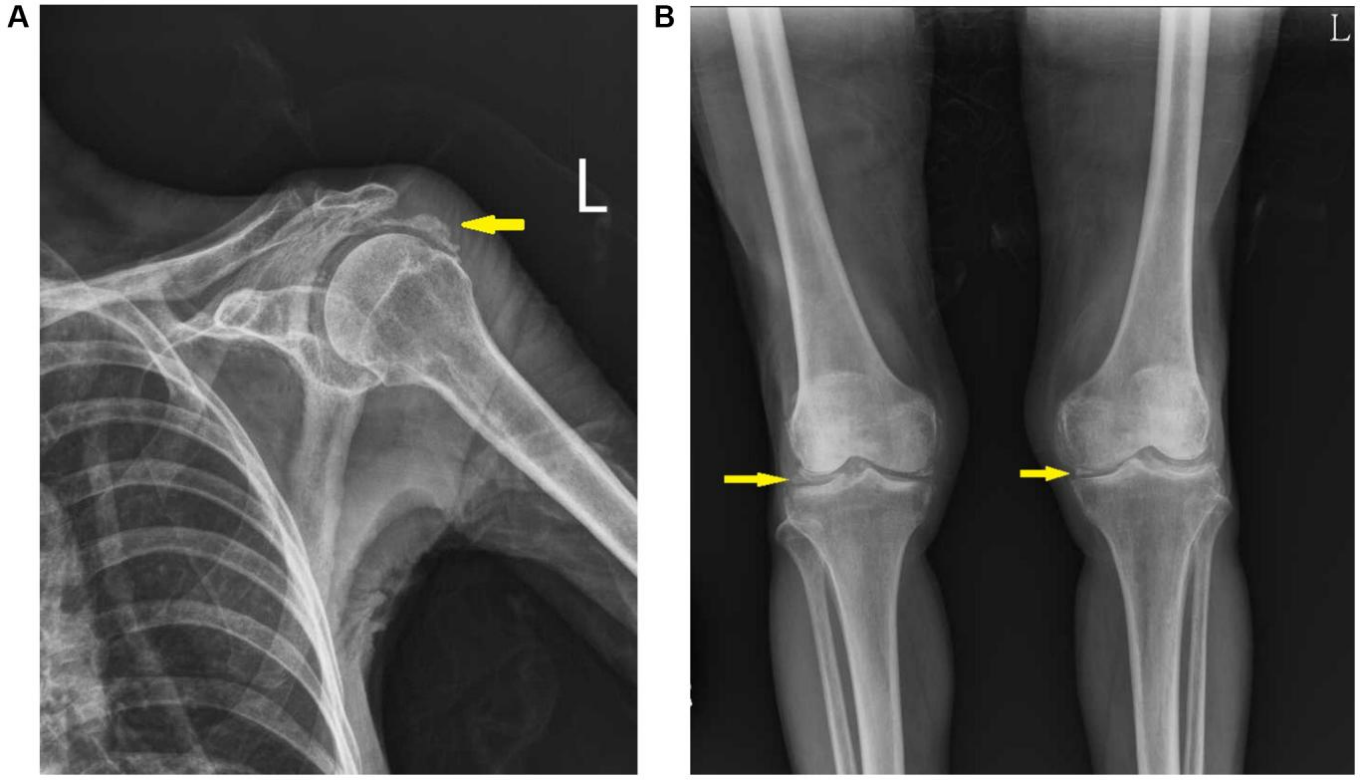
**Radiographic image of the shoulder and knee joints.** Calcifications were noted in the shoulder (**A**) and knee (**B**) joint space, compatible with chondrocalcinosis (yellow arrow).

Upon examination, the patient had a low body mass index (15.7 kg/m^2^, with a body height 143 cm and weight 32.1 kg) and profound kyphosis. Her blood pressure was 115/52 mmHg, with dry skin turgor. Although being alert, she was unable to organize her thoughts coherently. Other physical examinations were largely unremarkable. Serum biochemistry showed severe hypokalemia (2.0 mmol/L, reference range: 3.5–5.0 mmol/L), hypomagnesemia (1.1 mg/dL, reference range: 1.8–2.4 mg/dL), hyponatremia (124 mmol/L, reference range: 135–145 mmol/L), hypochloremia (87 mmol/L, reference range: 96–106 mmol/L), and normal total serum calcium (8.7 mg/dL) and creatinine (0.48 mg/dL). She denied using any diuretics, laxatives, β2- agonists or glucocorticoids. Blood gas analysis showed metabolic alkalosis (pH 7.44, reference range: 7.35–7.45; PaCO_2_ 47 mmHg, reference range: 32–48 mmHg; bicarbonate: 31.9 mmol/L, reference range: 24–28 mmol/L). Endocrinology tests revealed normal thyroid and adrenal function, with normal plasma renin and serum aldosterone levels. Urinalysis showed inappropriately elevated potassium excretion (trans-tubular K^+^ gradient 9.3, fractional excretion (FE) of potassium 15.0%), accompanied by elevated magnesium excretion (FEMg 14.0%) and hypocalciuria (daily excretion of 43.2mg Ca^2+^/creatinine: 0.06 mg/mg). Other serum and urine electrolyte findings are shown in Table 1. A coupled urinary sodium-to-chloride ratio (0.92, approximating 1) was also observed, suggesting non-gastrointestinal potassium loss [12]. Electrocardiogram disclosed a sinus rhythm with a prolonged QT interval (512 milliseconds). Radiographic examination showed calcific tendonitis and chondrocalcinosis affecting her left shoulder and knee joints (Figure 1). Arthrocentesis revealed inflammatory synovial fluid containing rod-shaped crystals, diagnostic of calcium pyrophosphate dihydrate crystal deposition disease. Bone mineral density examination revealed osteoporosis in the lumbar spines (L1 to L4, T-score ranging from −2.6 to −3.0). No steroid exposure was reported during the preceding two years of her current presentations. The constellation of symptoms, including salt craving, chronic volume depletion, glucose intolerance, and chronic hypokalemia and hypomagnesemia with hypocalciuria, pointed towards a tubulopathy characterized by an excessive loss of potassium and magnesium. To confirm the diagnosis of Bartter or Gitelman syndrome, targeted Sanger sequencing of the *SLC12A3* and *CLCNKB* genes was performed. Genomic DNA and cDNA were extracted from peripheral blood leukocytes, and specific regions of the *SLC12A3* and *CLCNKB* genes were amplified and sequenced. The analysis revealed a biallelic homozygous deletion mutation, c.2881-2AG, involving exon 24 of the *SLC12A3* gene (Supplementary Figure 2), confirming the diagnosis of GS.

**Table 1:** Summary of blood and urine laboratory values.

	**Value**	**Normal range**
Plasma		
Potassium, mmol/L	2.0	3.5–5
Chloride, mmol/L	87	96–106
Sodium, mmol/L	124	135–145
Magnesium, mg/dL	1.1	1.8–2.4
Calcium, mg/dL	8.7	8.6–10.1
Urea nitrogen, mg/dL	13	6–20
Creatinine, mg/dL	0.48	0.50–0.90
Osmolarity, mOsm/kgH_2_O	266	278–305
iPTH, pg/mL	24.3	15.0–65.0
pH	7.44	7.35–7.45
Bicarbonate, mmol/L	31.9	24–28
PaCO_2_, mm-Hg	47	32–48
Renin, pg/mL	27.5	1.8–59.4
Aldosterone, pg/mL	79.2	48.3–270
TSH, uU/mL	1.23	0.25–4.00
Free T4, ng/dL	1.40	0.89–1.79
Urine		
pH	6	5–8
Potassium, mmol/L	20	17–145
Chloride. mmol/L	75	20–300
Sodium, mmol/L	69	15–267
Magnesium, mg/dL	7.2	1.6–18.7
Calcium, mg/dL	1.9	6.8–21.3
Phosphate, mg/dL	7.1	40–136
Creatinine, mg/dL	32	28–217
Osmolarity, mOsm/kgH_2_O	285	850–1200
TTKG	9.3	<3
FE(Mg), %	14.0	2–4
FE(PO4), %	3.6	<5
Daily Ca urine loss, mg	43.2	100–250

Abbreviations: iPTH: intact parathyroid hormone; TSH: thyroid-stimulating hormone; Free T4: free thyroxine; TTKG: transtubular potassium gradient; FE(Mg): fractional excretion of magnesium; FE(PO4): fractional excretion of phosphate.

The patient was managed with a regimen of daily oral magnesium (approximately 50 mEq) and slow-release potassium (60 mEq) supplementation, alongside a liberal sodium intake. Treatment goals aimed to maintain flexible serum potassium levels above 3 mmol/L and magnesium levels over 1.4 mg/dL, with a focus on symptom relief and minimizing the pill burden. To mitigate risks such as gastrointestinal side effects, hypotension, and hypovolemia, non-steroidal anti-inflammatory drugs (NSAIDs), angiotensin-converting enzyme inhibitors, and potassium-sparing diuretics were avoided. A comprehensive care approach, involving an interdisciplinary team consisting of a geriatrician, nephrologist, dietician, and pharmacist, coordinated by a geriatric care practitioner, ensured the adherence to the treatment plan. During the ensuing two years, the patient experienced no further falls and maintained an active lifestyle.

## DISCUSSION

In this report, we described an old female with recurrent falls, thirst, and headache. Laboratory tests revealed hypokalemia, hypomagnesemia, and hypocalciuria without acquired disorders, raising the suspicion of inherited tubulopathy. Genotyping analysis showed a typical mutation involving *SLC12A3*, diagnostic of GS. Older patients are more prone to hypokalemia than their younger counterparts [13, 14] due to senescence of renal tubules and dietary alterations. The presentation of hypokalemia in older individuals often fails to trigger sufficient workup, including clinical history-taking and diagnostic test arrangement, to uncover potentially important diagnosis. Our case identified a two-base-pair deletion at nucleotide 2881–2 (del AG) in exon 24 of the *SLC12A3* gene, causing a frameshift from arginine at codon 959. This mutation, reported previously by our group, compromising the protein structure by eliminating the latter half of the intracellular carboxyl terminus essential for NCC functionality [15]. This case represents the oldest patient (83 years) at the time of GS diagnosis, marked by atypical presentations of recurrent falls, and demonstrates the life trajectory of patients with GS. Although genetic testing for GS is increasingly accessible, it remains costly and requires heightened clinician awareness, emphasizing the need for genetic screening in suspicious older cases.

The case study illustrates the diagnostic challenges posed by GS, particularly in older adults who present with late-life hypokalemia accompanied by subtle symptoms (such as short stature, frequent falls, chronic salt craving, volume depletion), underscoring the necessity for an in-depth differential diagnosis. GS is characterized by high symptom variability across various organ systems, with common manifestations including general symptoms (fatigue (82%) and dizziness (80%)), muscular symptoms (weakness (70%) and cramps (84%)), and renal symptoms (nocturia (80%) and polydipsia (64.6%)) [11]. Additionally, gastrointestinal symptoms such as constipation and abdominal pain occur in 16% of patients [11]. These symptoms, more prevalent than in age- and sex-matched controls, may contribute to diagnostic delays as late as 15 years [11]. An accurate diagnosis necessitates a clinical exclusion of other conditions including diuretic abuse, surreptitious vomiting, laxative abuse, and adverse drug effects from proton pump inhibitors and aminoglycosides. Biochemically, GS and Bartter syndrome (BS) can have similar presentations, with chronic hypokalemia, metabolic alkalosis, and renal potassium wasting. However, GS typically features hypocalciuria and low magnesium levels, in contrast to the near-normal plasma magnesium levels and childhood onset manifestations in BS. A study by Zelikovic et al. highlighted an identical homozygous mutation in the *CLCNKB* gene in 12 inbred kindred members (missense, R438H, exon 13), exhibiting overlapping features of both BS and GS [16]. This convergence of symptoms, late presentation in 58% of cases, hypomagnesemia in 42%, hypocalciuria in 42%, and varying degrees of polyuria/dehydration and hypercalciuria increases the challenge in distinctly categorizing affected patients into GS or BS groups [16]. Further complicating the diagnostic landscape, Lin et al. explored two unrelated Chinese families with five members exhibiting identical compound heterozygous NCC mutations (C2135A on exon 17 and base pair deletions at 2881-2 on exon 24) [15]. The intrafamilial phenotypic variability was striking, with males presenting severe symptoms akin to BS, including severe hypokalemia and symptomatic paralysis, whereas females displayed milder symptoms typical of GS, such as less severe hypokalemia, hypomagnesemia, and hypocalciuria [15]. This phenotypic variability may arise from the protective role of estrogen in regulating NCC function [17], contributing to less severe symptoms in females. Chen et al. demonstrated that renal excretion of sodium, chloride, and calcium is partly controlled by sex hormones via their regulation of the thiazide diuretic receptor (NCC) density [17]. Their research in Sprague-Dawley rats revealed significant differences in thiazide receptor density between sexes (twofold higher in females, *P* < 0.001) and the influences of orchiectomy (increasing receptor densities) and ovariectomy (decreasing) [17]. Nonetheless, establishing diagnosis for our patient is constrained by potential recall bias, with symptoms reported later in life but of unclear duration, and family members as the history reporting proxy may underestimate symptoms during the patient’s younger life period. These issues underscore the need for further large-scale research to explore the long-term outcomes of Gitelman syndrome in older populations.

In older adults, falls are a prevalent geriatric morbidity with potentially fatal consequences [18]. The etiologies for fall in older adults vary widely, encompassing both environmental and host-specific factors [19]. While neurodegenerative disorders are well-established origins, electrolyte disorders such as hypokalemia, hyponatremia and hypomagnesemia can also precipitate falls in older adults. Reduced extracellular potassium levels can affect nerve conduction velocity, alter evoked potential amplitude, and impair neuronal excitability [20]. Chronic hyponatremia, even at levels near normal range (around 131–134 mmol/L), can adversely affect bone quality over time, increasing osteoporotic fracture risk and fall susceptibility [21]. Patients with GS, akin to those receiving chronic thiazide therapy, often display increased renal calcium reabsorption, affecting bone remodeling [22]. Furthermore, hypomagnesemia in our patient may inhibit alkaline phosphatase activation and reduce calcium pyrophosphate solubility, thereby promoting crystal formation [2]. These joint changes, the presence of crystals and effusions, further impede post-fall recovery. Understanding these dyselectrolytemia–related factors is crucial for managing fall risks, enhancing functional independence, and reducing medical costs in older GS patients.

In GS, hyponatremia resulting from loss of NCC function is biologically plausible, given the increased incidence of hyponatremia in chronic thiazide users. However, coincident hyponatremia in our case remains infrequent in those with GS based on an extensive literature review [23]. It is plausible that the up-regulated aquaporin-2 expression may be less active due to the decreased medullary cyclic adenosine monophosphate generation in response to arginine vasopressin (AVP) in GS-like rats [24, 25]. The occurrence of hyponatremia in GS patients depends on the equilibrium between water intake and free water excretory capacity. A prior study reported that patients with genetically confirmed GS exhibited impaired osmole-free water clearance, as determined by inulin clearance (*P* < 0.01), compared to control participants [26]. Thiazide-associated hyponatremia often affects older, frail females, similar to our case [27, 28]. Among frail elderly with GS, lower effective circulatory volume may trigger the secretion of plasma AVP and impairs free water excretion. Our patient’s hyponatremia might arise from a mixture of altered free water excretion, an abnormal thirst response, and excessive AVP production due to the diminished effective circulatory volume.

Inherited GS is not amenable to cure and significantly affects patients’ quality of life (QOL) [2]. The degree of QOL reduction seen in those with GS is similar to that experienced by patients with congestive heart failure, diabetes, or coronary artery disease [11]. GS patients often have poorer daily activity, attributable to their perceived declining health, depression, and lack of motivation. This diminished QOL can be underestimated if our assessment solely relies on clinical symptoms. Common symptoms of impairing QOL include fatigue (82%), cramps (82%), and thirst (76%) [11]. Moreover, limited disease awareness with mis-diagnosis, combined with the burdensome pill numbers required to correct electrolyte imbalance, exacerbate QOL reduction in GS. Two-thirds of GS patients frequently seek emergency care, further increasing healthcare costs [11]. Timely diagnosis and management of GS in older adults are crucial to improve their QOL.

In conclusion, the diagnosis of GS should not be missed in the older adults, a population fraught with atypical disease presentations. We suggest that older adults with chronic and refractory hypokalemia with compatible laboratory features (hypomagnesaemia, hypocalciuria, and metabolic alkalosis) be screened for NCC dysfunction through genetic testing. An accurate diagnosis allows for targeted treatment strategies, reducing complications from electrolyte imbalance, better QOL and possibly strengthening patient-physician relationships.

### Availability of materials

This manuscript did not generate new materials.

## Supplementary Materials

Supplementary Figures
