# Supplementary material for: Recurrent falls as the presentations of Gitelman syndrome in an octogenarian
Source: Aging (Albany NY). 2025 Mar 4;17(3):872–80. doi: 10.18632/aging.206216 (PMC11984424; doi:10.18632/aging.206216)
Supplement: Supplementary Figures [file aging-17-206216-s001.pdf]

SUPPLEMENTARY FIGURES

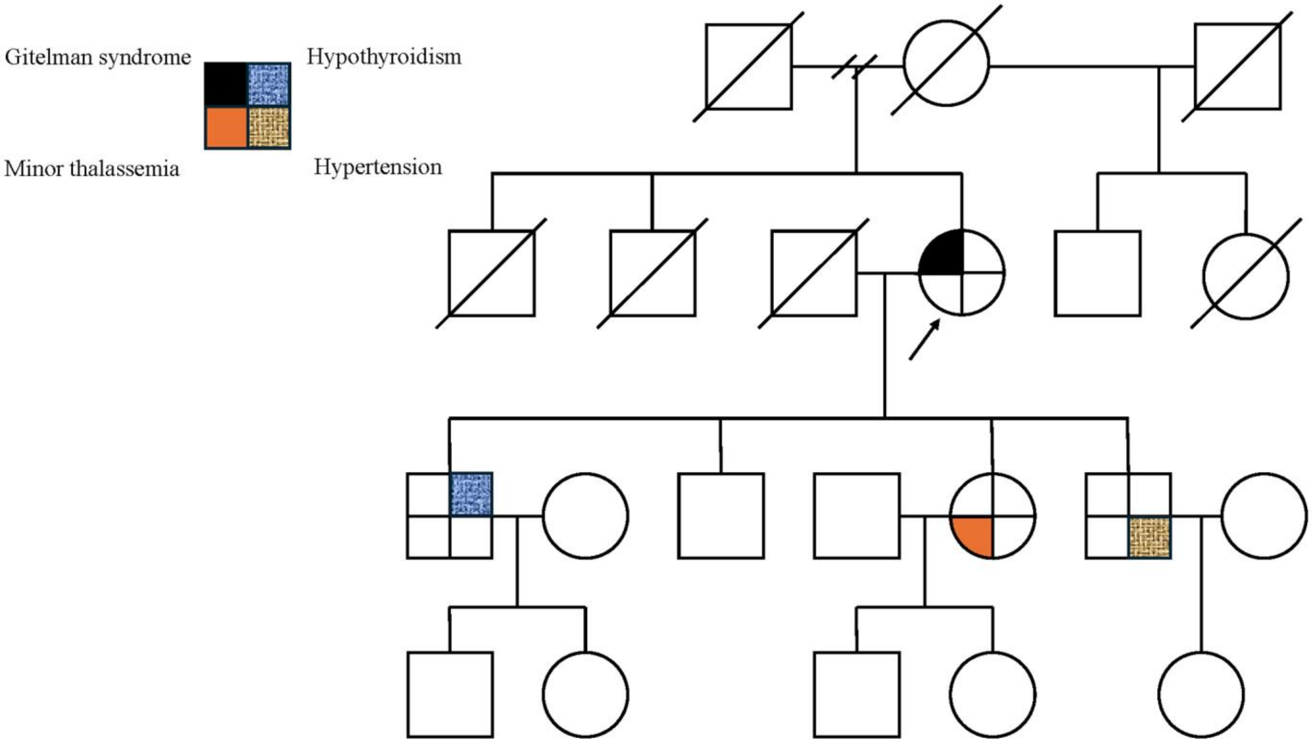

Supplementary Figure 1. Family pedigree.

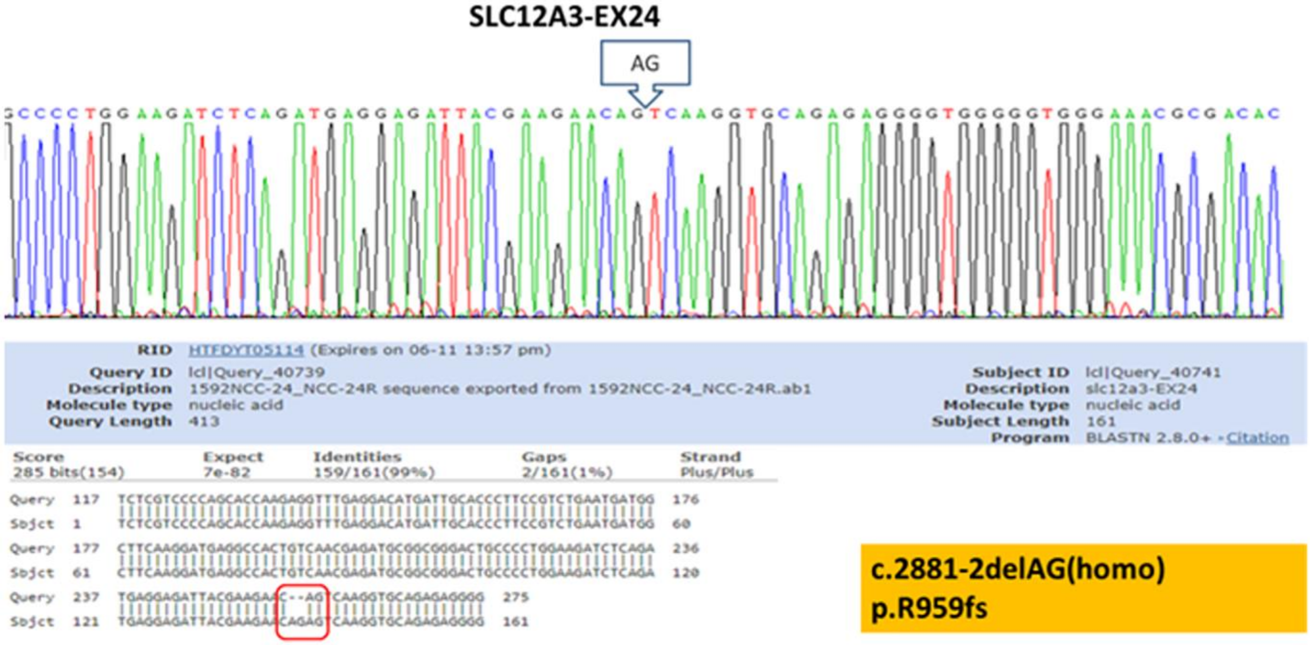

Supplementary Figure 2. *SLC12A3* genetic sequence analysis results of exon 24. Our patient carried a homozygous mutation of two base pair deletions at nucleotide 2881-2 (delAG).
